# Optical Coherence Tomography Angiography in Central Serous Chorioretinopathy

**DOI:** 10.1155/2015/134783

**Published:** 2015-11-08

**Authors:** Eliana Costanzo, Salomon Yves Cohen, Alexandra Miere, Giuseppe Querques, Vittorio Capuano, Oudy Semoun, Ala'a El Ameen, Hassiba Oubraham, Eric H. Souied

**Affiliations:** ^1^Department of Ophthalmology, University Paris Est Creteil, Intercity Hospital, 94000 Creteil, France; ^2^Department of Ophthalmology, Second University of Naples, Naples, Italy; ^3^Department of Ophthalmology, University Scientific Institute San Raffaele, Milan, Italy

## Abstract

*Purpose*. To analyze optical coherence tomography angiography (OCTA) findings in eyes with central serous chorioretinopathy (CSC) and to compare them with those obtained with multimodal imaging.* Methods*. A series of consecutive patients diagnosed with CSC, underwent OCTA and multimodal imaging, including spectral domain OCT, fluorescein, and indocyanine green angiography. OCTA images were performed at three main depth intervals: automatically segmented outer retina, manually adjusted outer retina, and automatically segmented choriocapillaris.* Results*. Thirty-three eyes of 32 consecutive patients were analyzed. OCTA showed 3 main anomalies at the choriocapillaris: the presence of dark areas (19/33 eyes) which were frequently associated with serous retinal detachment, presence of dark spots (7/33 eyes) which were frequently associated with retinal pigment epithelium detachment, and presence of abnormal vessels (12/33 eyes) which were frequently, but not systematically, associated with choroidal neovascularization, as confirmed by multimodal imaging.* Conclusions*. OCTA revealed dark areas and dark spots, which were commonly observed. An abnormal choroidal pattern was also observed in one-third of cases, even when multimodal imaging did not evidence any choroidal neovascularization. Abnormal choroidal vessels should be interpreted with caution, and we could assume that this pathological choroidal vascular pattern observed in many CSC cases could be distinct from CNV.

## 1. Introduction

Central serous chorioretinopathy (CSC) is a disorder characterized by episodes of macular serous retinal detachment (SRD), first described and characterized as a clinical entity with fluorescein angiography (FA) [1–3]. Several risk factors have been identified, such as glucocorticoid levels, type A personality, pregnancy, uncontrolled systemic hypertension, use of antibiotics, bone marrow or organ transplantation, infection of the respiratory tract, and infection by* Helicobacter pylori* [4–11]. The disease may present with different patterns: acute CSC, “persisting,” recurrent, and chronic CSC [12]. It usually occurs in young males but may be observed in older subjects of both genders. CSC is characterized by retinal pigment epithelium (RPE) changes that are easily observed on fundus autofluorescence (FAF) pictures, serous retinal pigment epithelium detachment (PED), and episodes of SRD [12]. FA usually shows areas of fluorescein leakage, often focal, with a smokestack or inkblot pattern in acute CSC [1–3], and more widespread in chronic stages, also called diffuse retinal pigment epitheliopathy [1, 3]. Indocyanine green angiography (ICGA) may show a hyperpermeability of the choroidal vessels, and sometimes diffuse dye leakage [13]. Optical coherence tomography (OCT), and especially enhanced-depth imaging (EDI) or swept source (SS) OCT, shows a thick choroid and may allow better characterizing the areas of PED and SRD [14, 15]. The disease may be complicated by type 1 choroidal neovascularization (CNV) [16]. However, this complication may be difficult to diagnose because chronic CSC and type 1 CNV share many signs, on both FA and OCT. CSC may also be associated with polypoidal choroidal vasculopathy (PCV), and both entities may be overlapped [12, 17].

Optical coherence tomography angiography (OCTA) is a new, noninvasive, nondye, depth-resolved technique, which may be used to investigate patients with CSC. The concept of OCTA is the detection of changes in blood flow in the vessels, in a static eye, without need for dye injection [18]. The amplitude of the signal returning from nonstatic features varies rapidly over time. When calculating the decorrelation of signal amplitude from repeated consecutive B-scans at the same cross section, a contrast between static and nonstatic tissues is created and generates a vascular decorrelation signal that enables visualizing 3-dimensional retinal and choroidal vasculature [19]. OCTA images may be studied by isolated segmentation in different vascular layers, thus allowing analyzing the vascular structure in detail with no darkening due to a staining or leakage [18–21]. The aim of this study was to analyze OCTA findings in eyes with CSC and to compare them with those obtained with multimodal imaging.

## 2. Methods

### 2.1. Study Population

A series of consecutive patients diagnosed with CSC at the University Eye Clinic of Creteil was examined. All patients with CSC were included, that is, patients with a clinical history of CSC and 6 months of visual impairment, patients previously treated or treatment-naive, and newly diagnosed, treatment-naive CSC patients. Patients with any associated, previous or concomitant, ophthalmological conditions that could confound the interpretation of clinical and imaging findings for the diagnosis of CSC (i.e., subretinal fibrosis, age-related macular degeneration, macular or retinal vascular disease, vitreoretinal disease or retinal surgery, and hereditary retinal dystrophy) were excluded from the study. This prospective clinical study was conducted in accordance with the French bioethical legislation and with the Declaration of Helsinki for research involving human subjects. It received the favorable opinion of the ethics committee of the France Macula Federation.

### 2.2. Study Protocol

Patients underwent a complete ophthalmic examination, including best-corrected visual acuity (BCVA) measurement with Early Treatment Diabetic Retinopathy Study (ETDRS) chart, slit-lamp examination, dilated-fundus biomicroscopy, and standardized imaging protocol, using Spectralis HRA + OCT (Heidelberg Engineering, Heidelberg, Germany), which included FAF, FA and ICGA, SD-OCT, and/or EDI-OCT. All patients also underwent OCTA using a commercially available RTVue XR Avanti with AngioVue (Optovue, Fremont, CA, USA). An incorporated software algorithm called Split-Spectrum Amplitude Decorrelation Angiography (SSADA) generates 3-dimensional en face angiograms through decorrelation of two merged consecutive orthogonal registration volumes automatically centered at the macula or manually centered at the lesion. Each acquired OCTA volume (3 × 3 mm) consisted of 304 × 304 A-scan in 2.6 seconds. The A-scan rate is 70,000 scans per second. Each orthogonal volume registration is corrected to minimize motion artifacts. Both orthogonal volume scans are combined with a motion correction technology (MCT) to create a 3-dimensional image of retinal and choroidal blood flow. A coregistered OCT B-scan allows the visualization of the retinal structure. RTVue XR Avanti has an automated segmentation at the superficial retinal capillary plexus, deep retinal capillary plexus, outer retina, and choriocapillaris [19–22]. The OCTA software was used to manually adjust the automated segmentation and its relative depth in the retina and choroid. OCTA images were analyzed at three main depth intervals: automatically segmented outer retina, manually adjusted segmentation between the RPE and 30 *μ*m beneath it, called manually segmented outer retina, in order to detect a blood flow in the RPE-Bruch membrane-choriocapillaris complex, and automatically segmented choriocapillaris.

Three trained OCTA users (Eliana Costanzo, Alexandra Miere, and Ala'a El Ameen) acquired the images and performed a qualitative analysis to determine CSC features. To assess the microvascular architecture, three retina specialists (Salomon Yves Cohen, Giuseppe Querques, and Eric H. Souied) analyzed a 3 × 3 scanning area at the three depths previously described. Patients with poor quality OCTA images (i.e., due to eye movement) were not included in the analysis. Moreover, eyes previously treated were analyzed separately from treatment-naive eyes.

Our analysis included descriptive statistics (using Microsoft Office Excel software; version 14.0, 2010, Redmond, USA) for demographics and main clinical data, as well as qualitative descriptions of the imaging findings.

## 3. Results

Thirty-three eyes of 32 consecutive patients (21 males, 11 females; mean age: 54.8 ± 10.4 years), diagnosed with CSC, were enrolled in this study. Mean BCVA was 0.23 ± 0.25 logMAR (range: 0–0.8 logMAR, corresponding to 20/20 to 20/125). Nine eyes (27.3%) had newly diagnosed treatment-naive CSC, while 24 eyes (72.7%) had a history of at least 6 months of visual symptoms in the affected eye. In 8 eyes (24.2%), CSC was associated with type 1 CNV.  Table 1 shows patient demographics and clinical characteristics.

For clarity, the whole population was analyzed first, with description of images obtained at different levels. In a second time, naive eyes were compared to previously treated eyes. In a third time, eyes with abnormal choroidal vessel patterns were described with more details, with an attempt of classification according to the pattern.

### 3.1. Systematic Analysis of Different Depth OCTA Images in the Whole Population

#### 3.1.1. Outer Retina

Automatically segmented outer retina OCTA images detected the presence of an abnormal flow in 6 eyes (18.2%) and no remarkable findings in 27 eyes (81.8%). The manually segmented area revealed the presence of an abnormal flow in 9 eyes (27.3%), while 24 eyes (72.7%) presented no particular abnormality.

#### 3.1.2. Choriocapillaris

**Figure 1:**
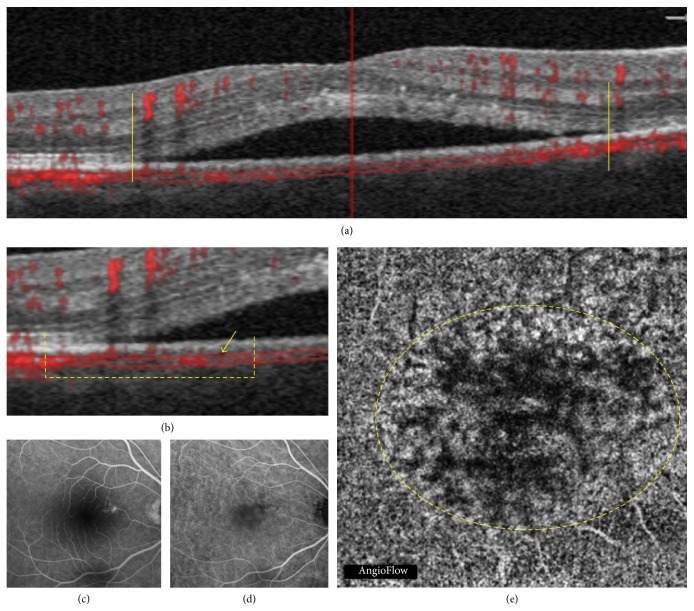
Acute central serous chorioretinopathy in a 48-year-old man. Correlation between optical coherence tomography (OCT), OCT angiography (OCTA), fluorescein angiography (FA), and indocyanine green angiography (ICGA). (a) OCT B-scan: the yellow vertical lines indicate the limits of the serous retinal detachment (SRD). (b) Detail of the OCT B-scan: the dashed yellow line shows the transition between the non-SRD and the SRD area; the yellow arrow indicates the area of flow reduction. (c) Late phase of FA: hyperfluorescent leaking point. (d) Early phase of ICGA: abnormal dilation of choroidal vessels. (e) OCTA at the choriocapillaris: the dashed yellow circle delineates an area of apparent blood flow reduction, called* dark area* in this study.

**Figure 2:**
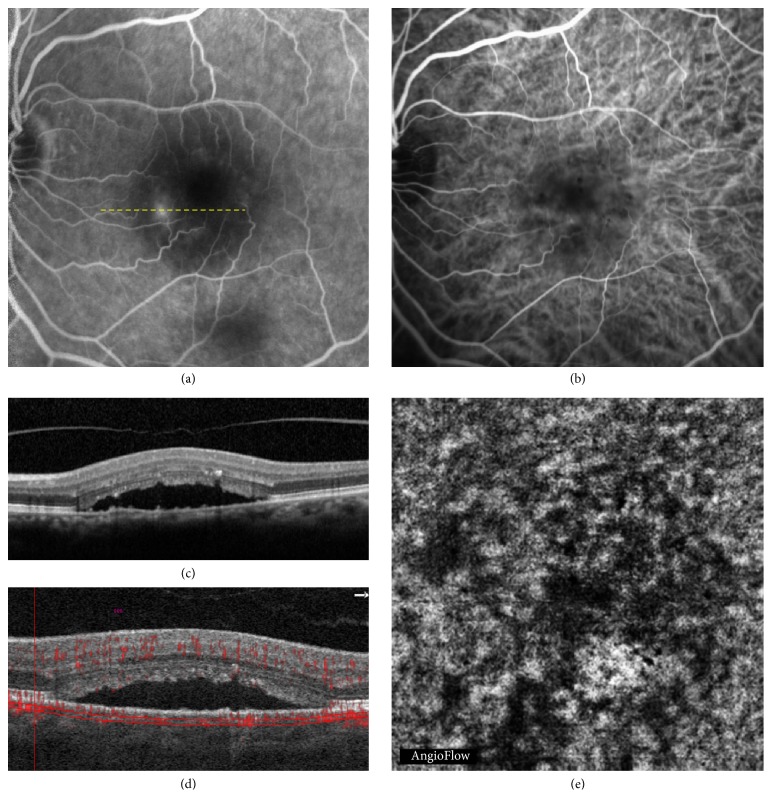
Chronic central serous chorioretinopathy in a 68-year-old woman. Correlation between fluorescein angiography (FA), indocyanine green angiography (ICGA), optical coherence tomography (OCT), and OCT angiography (OCTA). (a) Late phase of FA showing a typical leaking point; the yellow dashed line corresponds to the OCT scan shown in (c). (b) ICGA showing an abnormal dilation of choroidal vessels. (c) OCT B-scan showing a serous retinal detachment (SRD). (d) OCT B-scan showing the segmentation at the choriocapillaris shown in (e). (e) OCTA showing a dark area that corresponds to the SRD.

**Figure 3:**
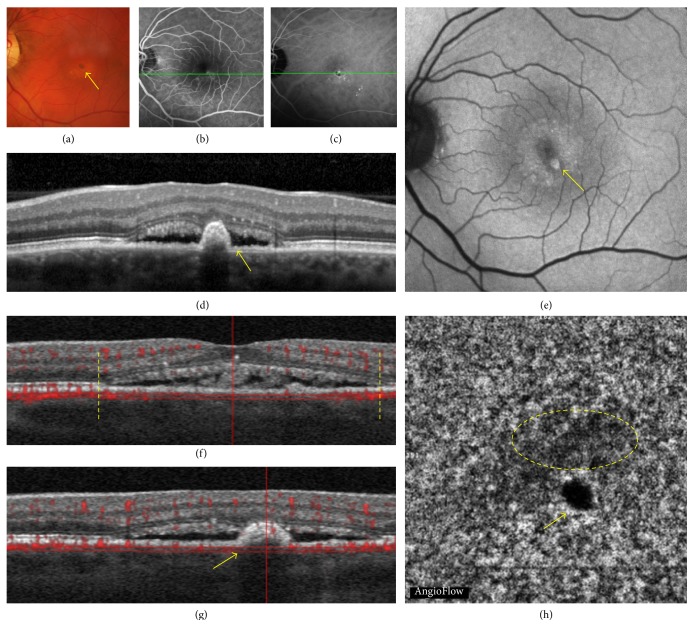
Recurrent central serous chorioretinopathy in a 61-year-old man. Multimodal imaging versus OCT angiography (OCTA). Multimodal imaging showing the correlation between color photography (a), fluorescein angiography (FA (b)), indocyanine green angiography (ICGA (c)), optical coherence tomography (OCT (d)), fundus autofluorescence (FAF (e)), and OCT angiography (OCTA (h)) with segmentation at the level shown on coregistered OCT B-scans ((f) and (g)). The color fundus photography showed pigmentary changes; the yellow arrow indicates a hyperpigmentation dot. Uneven leakage of dye ((b) and (c)). OCT B-scan showed a serous retinal detachment (SRD). Hyperautofluorescence with hyperautofluorescent parafoveal dots (arrow). Coregistered OCT B-scan showing the segmentation shown in (h): the yellow vertical lines indicate the limits of the SRD. Coregistered OCT B-scan at the level of the yellow arrow shown in (h): the yellow arrow indicates the small area of retinal pigment epithelium detachment. OCTA showed a moderately dark area delineated by a yellow dashed circle.

**Figure 4:**
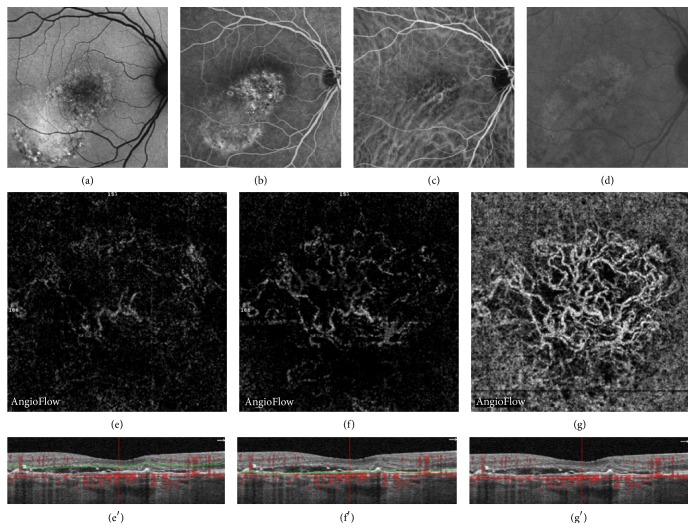
Central serous chorioretinopathy (CSC) complicated by choroidal neovascularization (CNV) in a 59-year-old woman. Multimodal imaging showing the correlation between fundus autofluorescence (FAF (a)), fluorescein angiography (FA (b)), indocyanine green angiography (ICGA (c) and (d)), and OCT angiography (OCTA (e), (f), (g)) with coregistered OCT B-scans showing the level of segmentation (e′, f′, g′). Chronic CSC with pigmentary changes and areas of hyperautofluorescence, uneven leakage of fluorescein, and dilated choroidal vessels ((a) to (c)) and presence of a choroidal neovascular membrane in the late phase of ICGA (d). Automatically segmented outer retina OCTA, showing a very moderate hyperdense signal (e). Manually segmented outer retina OCTA, detecting a hyperdense signal corresponding to a discrete blood flow (f). Automatically segmented choriocapillaris OCTA showing a hyperdense signal that highlights a tangled lesion with a typical neovascular network.

The choriocapillaris segmentation (from 30 *μ*m to 60 *μ*m beneath the RPE) was obtained for all patients except 2. It revealed three types of findings: dark areas, dark spots, and abnormal choroidal vessels. The dark areas corresponded to diffuse or focal, foggy, ill-defined, low-detectable flow areas in the choriocapillaris layer (Figures 1 and 2). The dark spots corresponded to black, single or multiple, well-delineated areas where no flow was detectable at the choriocapillaris. Dark spots could be observed alone or associated with dark areas (Figure 3). Abnormal choroidal vessels corresponded to distinct, well-delineated, high-flow, tangled pattern areas in the choriocapillaris layer (Figure 4) as well as an abnormal dilation of choroidal vessels. Results of OCTA were compared to those obtained with the coregistered OCT B-scan provided by the software. Results of the morphological correlation between dark areas and dark spots with OCT changes are shown in Table 2. There was a high correspondence between the presence of dark areas and SRD, which was observed in 17 out of 19 eyes.

### 3.2. Comparison of Naive and Previously Treated Eyes

Eyes were classified into two different groups. Group A included treatment-naive eyes (16 eyes, 48.5%) and group B included previously treated eyes (17 eyes, 51.5%). In group B, 8 eyes (47%) were treated with photodynamic therapy (PDT), 6 (35.4%) with intravitreal injection of anti-VEGF, and 3 (17.6%) with both therapies. In group A, the analysis of the automatically segmented outer retina did not find any abnormality. The analysis of the manually segmented outer retina found, in one case (6.25%), a hyperdense abnormal signal. The choriocapillaris analysis was normal in 2 cases (12.5%) but showed the presence of dark areas in 13 eyes (81.2%). More precisely, dark areas were isolated in 5 eyes (31.2%), associated with dark spots in 5 eyes (31.2%), and associated with abnormal choroidal vessels in 3 eyes (18.7%). An abnormal choroidal vessel, characterized by an abnormal dilation of choroidal vessels, was observed in one eye (6.7%). In group B, the analysis of the automatically segmented outer retina revealed an abnormal flow in 5 eyes (29.4%) and was normal in the remaining cases (70.6%). The manually segmented outer retina showed a hyperdense signal in 8 eyes (47%) and was normal in the remaining cases (53%). In this group, two patients were excluded from the analysis of the choriocapillaris layer because of an incorrect segmented slab automatically generated by the software. In the remaining 15 eyes, the choriocapillaris layer appeared normal in 1 case (6.7%) but showed a dark area in 4 eyes (26.7%), dark spots in 2 eyes (13.3%), abnormal choroidal vessels in 6 eyes (40%), and abnormal choroidal vessels with a dark area in 2 cases (13.3%). Comparison of the two groups showed that the presence of dark areas was significantly greater in group A (*p* = 0.029); there was no significant difference between groups regarding dark spots or abnormal choroidal vessels.

### 3.3. Description and Classification of Abnormal Choroidal Vessels

**Figure 5:**
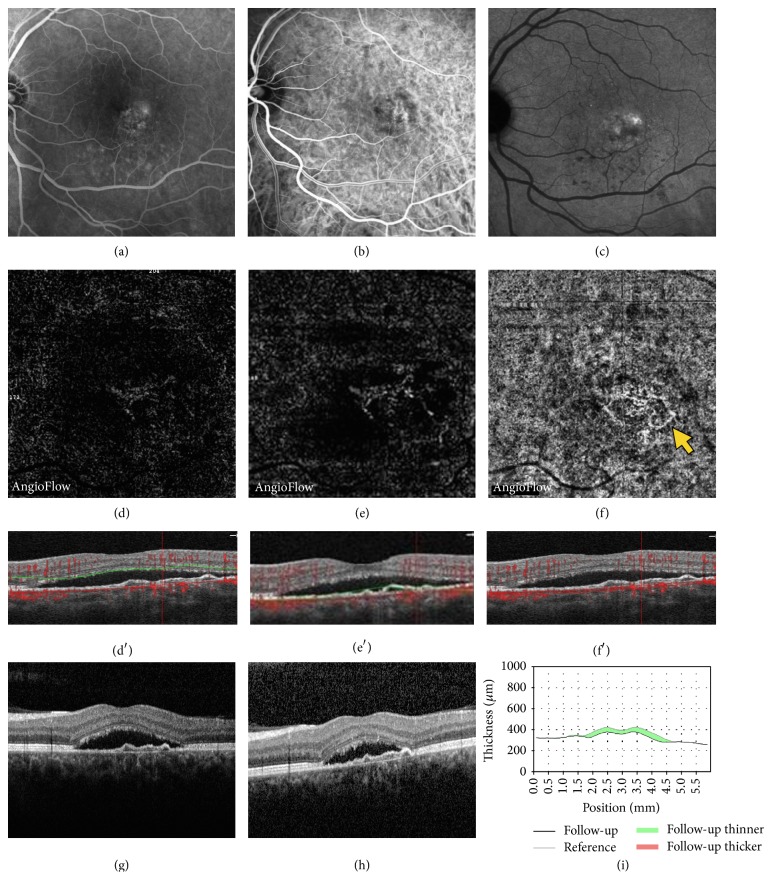
Central serous chorioretinopathy in a 52-year-old man with abnormal choroidal vessels on OCTA, but no evidence of CNV on multimodal imaging. Late phase of fluorescein angiography (FA (a)), early and late phase of indocyanine green angiography (ICGA (b) and (c)), OCTA (d, e, f), and coregistered OCT B-scan (d′, e′, f′). Late phase of ICG revealed faint hyperfluorescent points, which are usual features of CSR [13] but no clear evidence of neovascular choroidal membrane. Automatically segmented outer retina OCTA image did not show any evidence of hyperdense abnormal signal (d). Manually segmented outer retina OCTA image showing a moderate abnormal hyperdense signal (e). Automatically segmented choriocapillaris OCTA image showing an abnormal choroidal vessel (arrow) with a tangled pattern lesion. OCT revealed mild flat irregular PED and SRD (d′, e′, f′, g). This patient was not treated. After one-month follow-up, the SRD spontaneously decreased (38 microns), arguing in favor of absence of neovascular lesion (h, i).

Twelve eyes out of 33 showed an abnormal choroidal vessel pattern at the choriocapillaris (Table 3). In a recent study, we described patterns of CNV inside fibrotic scars as “pruned tree” defined as an irregular, filamentous flow inside the neovascular network and “tangled pattern” as an abnormal, high-flow, frequently interlacing vascular network [23]. One eye showed a pruned tree pattern. One eye showed an abnormal diffuse dilation of choroidal vessels without distinct pattern. The most common finding was a tangled pattern, observed in 10/12 eyes. Tangled pattern could be divided in two kinds of lesions: a “ball of wool-like” pattern or an indistinct tangled pattern. Atypical “ball of wool-like” tangled pattern was observed in 5/10 eyes. All these 5 eyes harboring a “ball of wool-like” pattern had typical CNV on multimodal imaging (Figure 4). In the other 5/10 eyes, an indistinct tangled pattern with high-flow abnormal choroidal vessels was observed. Multimodal imaging of these 5 eyes with indistinct tangled pattern confirmed the presence of CNV in 2/5 eyes. In the remaining 3/5 eyes with indistinct tangled pattern, multimodal imaging and follow-up of the patient did not show evidence of CNV (Figure 5).

## 4. Discussion

This study described a series of consecutive patients with CSC, whatever the stage of the disease, analyzed by OCTA, and aimed to compare the images obtained with those obtained with other imaging modalities. Main findings were the presence of dark areas which were frequently associated with SRD, presence of dark spots which were frequently associated with PED, and presence of abnormal vessels at the choriocapillaris which were frequently, but not systematically, associated with CNV based on multimodal imaging.

Three studies, one case report and 2 case series, have previously reported OCTA findings in CSC patients. All studies have focused on the diagnosis of associated CNV. They demonstrated that OCTA was able to detect the distinct neovascular network, typical of CNV, in 58% of eyes with chronic CSC [20]. Another study has shown the very high sensitivity and specificity (100%) of OCTA for the diagnosis of associated CNV [21]. The case report has shown a focal choriocapillaris defect related to the RPE focal loss [22]. In our series, 8 CSC complicated by CNV were included. Our findings confirmed that, in 100% of cases, OCTA was able to show an abnormal choroidal vessel pattern at the choriocapillaris as well as detect an abnormal flow in the manual outer retina segmentation. However, interestingly, this study also revealed 3 cases of false positive, that is, which had an abnormal choroidal vessel pattern at the choriocapillaris, but no typical features of neovascular membrane on multimodal imaging. Thus, this study suggests that interpreting abnormal choroidal vessels at the choriocapillaris should be made with caution in CSC patients and should not systematically be considered as CNV. In other words, our study compared OCTA and multimodal imaging findings, including FA, ICGA, and SD-OCT. Due to our experience in the field of CSC, we could ascertain the absence of CNV in some cases, based on multimodal imaging. However, we could assume that the irregular choroidal pattern revealed a pathological choroidal vasculature that could correspond either to abnormally dilated choroidal vessels or to CNV.

This study disclosed two previously unreported findings in CSC patients: dark areas and dark spots at the choriocapillaris. The choriocapillaris has a fine, densely packed, honeycomb-like microvasculature at the central fovea and this aspect changes when moving toward the optic nerve or the periphery [24]. In our series, a rarefaction area was observed at the choriocapillaris, and the term “dark area” was used to describe it. These findings could, however, be an artifact due to a process of light attenuation or altered signal returned by the SRD, flat irregular PED, or outer segment photoreceptor elongation or a combination of all these features. But dark areas could also correspond to a flow void, due to a focal atrophy of the choriocapillaris secondary to compression by the enlarged vessels from the outer choroid, as previously suggested in CSC [25]. Interestingly, OCTA also showed areas without detectable flow that were called dark spots in our study. Focal flow reductions in the choriocapillaris layer could be one of the causes of dark areas.

In conclusion, OCTA appears to be a promising technique because it avoids the burden of intravenous injections of dye that may sometimes be complicated by serious side effects. However, interpreting images needs a learning curve of knowledge. This report aimed to describe OCTA findings observed in CSC and to correlate them with other imaging modality findings. It showed dark areas and dark spots as common findings observed at the choriocapillaris in eyes with CSC. It also revealed the presence of abnormal dilated choroidal vessels at the same level that were frequently but not systematically associated with CNV, raising the question of OCTA imaging interpretation and specificity. Further studies are needed to confirm these preliminary results and assess the usefulness of this noninvasive tool in the diagnosis and management of CSC.

## Figures and Tables

**Table 1:** Main demographical and clinical findings of study patients.

Patient, *n*	33
Male, *n*	21
Female, *n*	11
Age, y (mean ± SD)	54.8 ± 10.4
BCVA logMAR, (mean ± SD)	0.23 ± 0.25
Acute CSC (first diagnosis), *n of eyes*	9 (27.3%)
Chronic CSC, *n of eyes*	24 (72.7%)
Previous treatment in chronic CSC	
PDT	8 (33.3%)
IVT	6 (25%)
PDT + IVT	3 (12.5%)
No treatment	7 (29.2%)

*n*: numbers, y: years, SD: standard deviation, CSC: central serous chorioretinopathy, PDT: photodynamic therapy, and IVT: intravitreal injection of Antivascular Endothelial Growth Factor.

**Table 2:** Automatic segmented choriocapillaris OCTA features in CSC patients.

Dark area, *n*	19
SRD, *n*	17
Irregular flat, *n*	7
Hyperreflective deposit, *n*	4
OS elongation	11
Dark spot, *n*	7
PED (irregular flat), *n*	5
Hyperreflective deposit, *n*	2
Pigment migration, *n*	2
Abnormal vessels, *n*	12
Dilation, *n*	1
Indistinct tangled, *n*	5
Tangled “ball of wool,” *n*	5
Pruned tree, *n*	1
No abnormalities, *n*	3

*n*: numbers of eyes; dark area: 19, dark spot: 7, abnormal choroidal vessel: 12, and no abnormalities: 3.

SRD: serous retinal detachment; PED: pigment epithelial detachment; OS: photoreceptor outer segments.

**Table 3:** Comparison between abnormal choroidal vessel at the choriocapillaris level and multimodal imaging evidence of choroidal neovascularization (CNV).

Eye	Abnormal choroidal vessel pattern	Multimodal imaging features
#1	Typical tangled, “ball of wool”	CNV
#2	Typical tangled, “ball of wool”	CNV
#3	Indistinct tangled pattern	CNV
#4	Typical tangled, “ball of wool"	CNV
#5	Indistinct tangled pattern	No CNV
#6	Indistinct tangled pattern	No CNV
#7	Typical tangled, “ball of wool"	CNV
#8	Pruned tree pattern	CNV
#9	Indistinct tangled pattern	CNV
#10	Indistinct tangled pattern	No CNV
#11	Abnormal dilation	No CNV
#12	Typical tangled, “ball of wool”	CNV
